# Stress Deflection Effect and Rockburst Mechanism in Staggered Roadways Beneath “L-Shaped” Residual Pillar

**DOI:** 10.3390/s26041173

**Published:** 2026-02-11

**Authors:** Qiang Lu, Jiancheng Jin, Siyuan Gong, Hui Li, Rupei Zhang, Bingrui Chen, Ying Qu, Zonglong Mu

**Affiliations:** 1 School of Mines, China University of Mining and Technology, Xuzhou 221116, China; ts20020041a31@cumt.edu.cn (Q.L.);; 2 Huaneng Qingyang Coal and Electricity Co., Ltd., Qingyang 745000, China; 3 Key Laboratory of Ministry of Education on Safe Mining of Deep Metal Mines, Northeastern University, Shenyang 110819, China; 4 Huating Coal Mine, Huating Coal Industry Group Co., Ltd., Huating 744100, China

**Keywords:** rockburst, stress deflection, residual coal pillar, source mechanism, numerical simulation

## Abstract

**Highlights:**

**What are the main findings?**

- We proposed a novel method for correcting the vertical height of rockburst hypocenters based on the moment tensor force mechanism.
- The predominant type of source rupture under the influence of residual coal pillars is compressive fracturing.

**What are the implications of the main findings?**

- The “L-shaped” high-stress structure formed by residual coal pillars and its stress deflection effect are the primary causes of rockbursts.

**Abstract:**

Frequent rockbursts in staggered roadways beneath residual coal pillars pose a critical challenge for the slice mining of ultra-thick coal seams. Taking the LW250101-2 of Huating Coal Mine as a case study, this paper systematically reveals the stress evolution laws and rockburst mechanism induced by irregular residual pillars by integrating microseismic (MS) monitoring, moment tensor inversion, and numerical simulation. First, source mechanism inversion analysis elucidated that compressive-shear failure of coal pillars was the dominant rupture mode in five of the eight recorded rockburst events. Second, numerical simulations demonstrate that the width of the left wing and the thickness of the right wing of the “L-shaped” coal pillar structure are the key geometric factors controlling rockburst risk; larger dimensions correlate with more intense stress concentration and higher-energy MS events. Moreover, the stress deflection effect of “L-shaped” coal pillars causes the haulage gateway of the LW250101-2 to remain in a state of stress accumulation, increasing its susceptibility to rockburst. Finally, a synergistic prevention system consisting of deep-hole roof blasting, large-charge coal blasting, and ultra-deep large-diameter boreholes was implemented. Field monitoring confirms that these measures dissipated high-stress concentrations, reduced rockburst frequency to zero and ensured safe mining.

## 1. Introduction

Rockburst is a dynamic failure phenomenon in mining characterized by the instantaneous release of elastic energy from coal and rock masses. With the continuous increase in mining depth, intensity, and complexity of mining conditions, the risk of rockburst disasters has become increasingly severe [1,2,3,4]. The inducing factors are diverse and complex, among which coal seam thickness is one of the significant influencing factors. In China, thick coal seams account for approximately 44% of the total reserves, contributing about 45% of the total raw coal production [5]. The thicker the coal seam, the greater the accumulated elastic strain energy and the higher the likelihood of rockburst occurrence. To mitigate the impact of coal seam thickness on rockburst, layered mining is generally adopted for ultra-thick coal seams. The mining of the upper layer in ultra-thick coal seams provides ample pressure relief space for the lower seams [6,7], significantly increasing the space for overlying strata movement and effectively releasing the load from strata activities. This ensures pressure relief for the safe extraction of lower coal seams. Therefore, for rockburst prevention and control under thick and ultra-thick coal seam mining conditions, the layered mining method is highly applicable and demonstrates the most significant effectiveness [8,9,10].

Due to historical mining practices and the need to support overlying strata loads, a certain width of coal pillars (overlying residual coal pillars) is typically left after the extraction of the upper layer to ensure the stability of the underground space post-mining [11]. However, coal pillars are primary zones of stress concentration, and the residual pillars—particularly those of irregular shape—often exert a pronounced influence on rockburst risk [12,13]. Particularly when wide residual pillars exist in the overlying goaf, extensive stress concentration occurs in the pillar area, and this concentrated stress propagates downward through the floor strata of the upper layer [14,15,16]. If the layout of the lower layer working face is improper, the face is subjected to the combined effects of mining-induced stress and the floor stress from the overlying residual pillars. This makes the coal and rock masses in the affected area highly prone to instability, deformation, severe roof convergence, floor heave, and other intense ground pressure manifestations, seriously restricting safe and efficient mine production.

To address this issue, the staggered layout method is commonly adopted, where the extraction roadways of the lower layer are positioned beneath the goaf to reduce rockburst risk and ensure production safety [17]. In such cases, the extent of influence of overlying residual pillars from thick and ultra-thick coal seam layered mining on the lower layer’s extraction roadways is closely related to the offset distance of the roadway layout. Generally, a larger offset distance between the lower layer extraction roadway and the overlying residual pillars results in less disturbance from the high stress of these pillars, leading to greater roadway stability. However, considering the rationality and feasibility of roadway layout, the offset distance between the lower and upper layer roadways is usually kept within a small range. This approach not only reduces the impact of peak stress in the pillar zone but also allows for effective support in relatively intact surrounding rock, achieving an optimal balance between the mechanical environment and construction conditions. Xu et al. [18] determined the distribution pattern of stress under overlying residual pillars through numerical simulation and theoretical analysis, designing the optimal position for roadway layout. Li et al. [19] applied soil mechanic principles and numerical analysis methods to study the stress transfer law in overlying residual pillars, concluding that these pillars generate high pressure and large suspended areas. Qi et al. [20], based on plastic slip line field theory and limit equilibrium theory, combined with numerical simulation methods, investigated the bearing failure range of the floor under the concentrated load of section pillars and its impact on the stability of surrounding rock in roadways. Li et al. [21] studied the disturbance evolution mechanism of overlying residual section pillars on floor stress before and after the extraction of the underlying coal seam, according to the disturbance propagation law of floor stress from these pillars, and calculated and analyzed the disturbance width range for mining the underlying coal seam below the residual pillar area. Vu [22] used UDEC software to study the stability of coal pillars when mining each layer close to each other based on the geological conditions of Nui Beo Coal Mine, Vietnam. Le et al. [23]. conducted an in-depth analysis of the stress evolution process induced by longwall mining through the UDEC numerical simulation method. Their study revealed the re-concentration mechanism of horizontal stress at the fixed end of the cantilever beam during periodic weighting, as well as the fracture pattern characterized primarily by tensile failure in the roof and shear failure in the floor. Using FLAC3D numerical simulation, Le et al. [24] investigated the distribution pattern of floor stress beneath the upper coal pillar in close-distance coal seam mining at Thong Nhat Coal Mine in Vietnam and its impact on the stability of the lower roadway. Sun [25] used FLAC3D and PFC2D numerical simulations to study the effect of the horizontal distance between the lower seam roadway and the overlying coal pillar on surrounding rock control and reasonable roadway layout, clarifying the influence mechanism of overlying pillar width on stope stress.

While existing research has achieved significant results in understanding stress distribution patterns in overlying coal pillars and optimizing roadway layouts, most studies focus on regular coal pillars. No research has been conducted on the stress deflection characteristics induced by irregular coal pillars resulting from unreasonable mining layout, nor has the moment tensor inversion method coupled with source depth correction been integrated to analyze the mechanism of rockburst occurrence. Mining operations are extremely complex engineering activities, where the movement of overlying strata and the stress distribution in surrounding rock continuously change as coal is extracted. Under such conditions, even if the lower-seam roadway is located within the influence zone of protective seam mining, it still retains the potential for rockburst occurrence. Furthermore, the mechanism of rockburst in this context is complex and requires specific analysis under particular conditions. In view of this, this study takes the staggered-seam roadway under the influence of an overlying wide residual coal pillar in a typical ultra-thick coal seam mining area as its engineering background. A systematic investigation is conducted by comprehensively employing multiple methods, including field measurements, microseismic (MS) analysis, theoretical calculations, and numerical simulations. The aims are: (1) to clarify the dominant controlling factors of rockburst and the source rupture mechanism under complex geological conditions by integrating numerical simulations and the moment tensor-based source depth correction method; (2) to reveal the evolution law and stress deflection characteristics of the surrounding rock stress field under the influence of an overlying irregular residual coal pillar; (3) to propose and apply a technical system for rockburst prevention and control in staggered-seam roadways based on stress regulation. This research will not only deepen the understanding of the rockburst mechanism in complex stress environments and enrich rockburst theory but also provide scientific and effective disaster early warning and prevention guidance for mines with similar conditions. It holds significant theoretical importance and engineering application value for ensuring the safe mining of deep ultra-thick coal seams in China.

## 2. Engineering Background

### 2.1. Working Face Overview

#### 2.1.1. Working Face Mining Design

**Figure 1:**
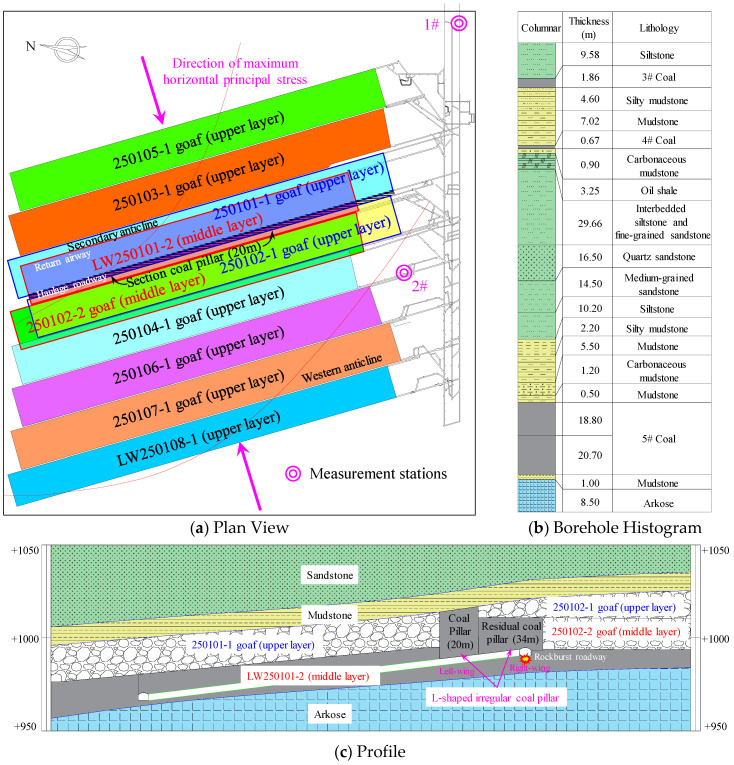
Layout diagram of the working face.

Huating Coal Mine is located in the mid-southern section of the eastern wing of the Huating Coalfield syncline in Gansu Province. It is 3.2 km in strike length and 3.6 km in dip width. The mine primarily exploits the No. 5 coal seam, which has a dip angle ranging from 4° to 12°. The seam thickness varies from 17.6 m to 48.2 m, with an average thickness of 37.51 m, classifying it as an ultra-thick coal seam mined in three slices. Among these, the LW250101-2 is the second middle-slice working face in the 2501 mining district of the No. 5 coal seam. It has a designed minable length of 1800 m and a width of 200 m. A secondary anticline passes through the middle of the coal seam, but overall, the coal seam occurrence within the mining area of this working face is relatively gentle. Its northern boundary is the mine boundary, the southern part is protected by main roadway coal pillars, the western part is the goaf of the LW250102-2, and the eastern part is solid coal. The roof consists of the upper-slice goafs of LW250101-1 and LW250102-1, between which a 20 m-wide section pillar was left. Furthermore, due to the unequal mining heights of the two upper-slice working faces and the staggered layout of the LW250102-2, an 34 m-wide residual coal pillar remains between the LW250102-2 goaf and the 20 m section pillar. These two pillars together form an overlying “L-shaped” irregular coal pillar (its geometric irregularity and stress distribution patterns are discussed in detail in Section 5.1) above the LW250101-2 haulage gateway. A schematic diagram of the working face layout is shown in Figure 1.

#### 2.1.2. Coal-Rock Bursting Liability and In Situ Stress Measurement Results

The immediate roof strata of the LW250101-2 consist mainly of mudstone and siltstone, while the floor strata are primarily composed of feldspathic sandstone. Based on coal burst tendency tests, the uniaxial compressive strength of the upper-slice coal is 13.7 MPa, indicating strong burst tendency; the middle-slice coal has a strength of 5.78 MPa, indicating weak burst tendency; and the lower-slice coal has a strength of 7.40 MPa, also indicating weak burst tendency.

To understand the in situ stress distribution at Huating Coal Mine, two measurement points (1#, 2#) were arranged in the No. 5 coal seam, and the stress relief method was used for in situ stress testing. The results are shown in Table 1. The results indicate that the maximum principal stress is the horizontal principal stress, oriented perpendicular to the coal seam strike, characterizing a typical horizontal tectonic stress field.

### 2.2. Characteristics of MS Events Distribution and Rockburst Manifestations

**Figure 2:**
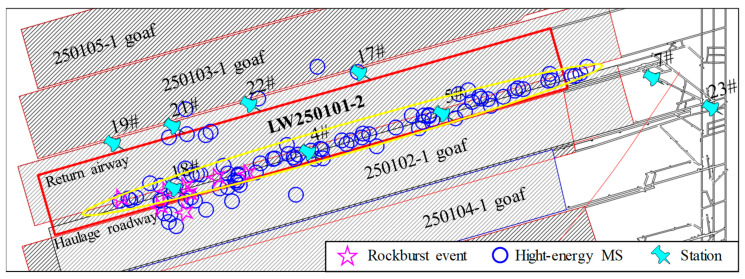
Spatial projection map of high-energy MS events distribution.

**Figure 3:**
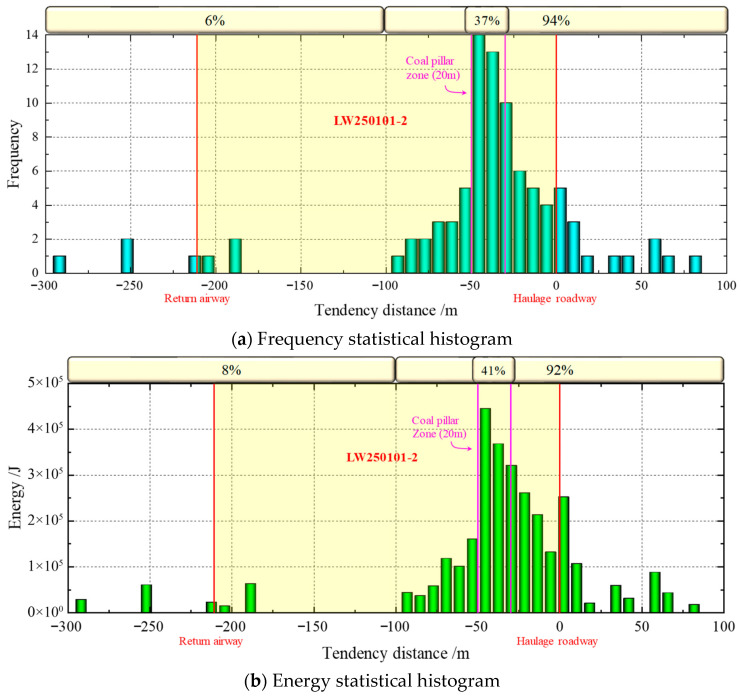
Statistical histogram of high-energy MS distribution along the working face tendencies. The yellow area represents LW250101-2; the left red line indicates the return airway; the right red line denotes the haulage roadway; and the pink-line area marks the coal pillar zone (20 m wide).

The spatial distribution of MS events can indirectly reflect the distribution of the excavation-disturbed stress field, indicating the damage and deterioration of the rock mass to a certain extent and reflecting the overall trend of large deformations in the rock mass and rock fractures [26,27]. MS events with higher energy levels signify stronger energy release and are more likely to induce severe rockburst risks [28]. Huating Coal Mine introduced and installed a 32-channel SOS MS monitoring system. This system is used to monitor MS events activity in the mine’s excavation areas in real-time, determining their source locations and energy, thereby providing a basis for rockburst monitoring and prevention. The SOS MS monitoring system collects signals from MS and rockburst events and solves for their source parameters to obtain the source location and energy. MS events typically occur during the full mining stage of the working face, and their disaster-inducing effects on roadways are also generated during this period [29]. Accordingly, the source locations of all high-energy microseismic events (energy greater than 1 × 10^4^ J) [30] and rockburst events recorded during the production of the 250101-2 working face were projected onto a plan view, as shown in Figure 2. Simultaneously, to further quantify their spatial distribution characteristics, the energy and frequency of high-energy MS events were statistically analyzed along the working face’s dip direction, as shown in Figure 3.

As shown in Figure 2 and Figure 3, high-energy MS events are distinctly concentrated in a banded zone on the side of the LW250101-2 haulage gateway. Their energy levels and frequencies are generally higher than those on the opposite side. Along the roadway’s strike direction, these events exhibit a gradient characteristic of being “denser in the front and sparser in the rear” while displaying a typical normal distribution along the dip direction. The area of the 20 m wide coal pillar serves as the core zone for high-energy MS concentration, accounting for as high as 41% and 37% of the total frequency and energy, respectively. Meanwhile, the source locations of all occurred rockburst events are also situated on this side, showing significant spatial overlap with the high-energy MS concentration zone. This coupling relationship indicates that the coal-rock mass on the haulage gateway side is in a state of high stress and high energy accumulation. It also verifies the correlation between the spatiotemporal evolution of high-energy MS activity and rockburst occurrence.

**Figure 4:**
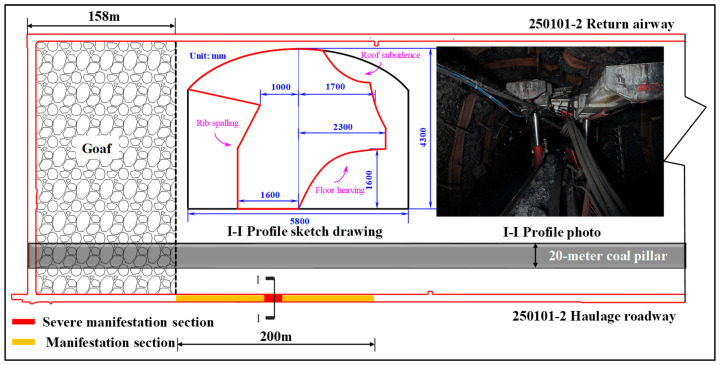
Manifestation and damage of rockburst (taking the “3.22” rockburst event as an example).

During the mining period of the LW250101-2, more than ten rockburst events occurred. According to field investigations, the damage was exclusively located within the haulage roadway, primarily manifesting as floor heave. A minority of these rockburst events were accompanied by rib spalling, roof sagging, and damage to support and transportation equipment. A schematic diagram illustrating the damage is presented in Figure 4.

## 3. Source Rupture Mechanism of Rockburst

### 3.1. Source Depth Correction Method Based on Moment Tensor Inversion

Different rockburst events exhibit varying energy release magnitudes and disaster-causing effects due to differences in their triggering causes and source rupture mechanisms. Understanding the rupture mechanisms of mining-induced seismic events is therefore significant for distinguishing between different types of rockburst events. The seismic moment tensor represents a generalized concept utilized to describe the source equivalent force and provides a quantitative expression of the source radiation pattern and intensity [31]. Utilizing the moment tensor allows for the linear inversion of source parameters. The source mechanism solution obtained from this inversion can effectively identify the rupture mode of the source, thereby providing a crucial basis for the prevention and control of rockbursts. Under the assumption of simplifying the surrounding rock as a homogeneous medium, the theoretical amplitude of the P-wave first motion displacement generated on a sensor at a far-field position x by a source with a moment tensor mechanical mechanism *M* = *M_pq_* (*p* = 1.3, *q* = 1.3) is given by:

(1) $$u_{n}\left(x\right)=\sum_{p=1}^{3}\sum_{q=1}^{3}\frac{\gamma_{n}\gamma_{p}\gamma_{q}}{4\pi \rho \alpha^{3}}\frac{1}{r}M_{pq}$$

where *ρ* is the density of the surrounding rock; *α* is the propagation velocity of the P-wave; *r* is the distance between the seismic source *ξ* and the sensor *x*, $r=\left|x-\xi \right|$; the direction vector $\gamma_{i}=\left(x_{i}-\xi_{i}\right)/r,\;i=1\ldots 3;\;n=1\ldots 3$, representing three spatial directions (two mutually perpendicular horizontal directions and one vertical direction). When only a vertical single-component sensor is used, *u_3_*(*x*) represents the peak displacement of the P-wave first motion in the vertical component. After processing the recorded waveforms from the sensors and obtaining the peak displacements of the P-wave first motions from at least six directions, the moment tensor *M* can be solved using the linear least squares method. The moment tensor *M* can be further decomposed into three fundamental components: the isotropic component (*ISO*), the double-couple component (*DC*), and the compensated linear vector dipole component (*CLVD*):

(2) $$M=M_{ISO}E_{ISO}+M_{DC}E_{DC}+M_{CLVD}E_{CLVD}$$

In the formula, *M_ISO_*, *M_DC_*, and *M_CLVD_* are the coordinates of the *ISO*, *DC*, and *CLVD* components, respectively, in the three-dimensional source-type space; *E_ISO_*, *E_DC_*, and *E_CLVD_* are the base tensors for the *ISO*, *DC*, and *CLVD* components. The calculated percentage of each component plays a critical role in the identification of the source rupture type:

(3) $$\left[\begin{array}{l} C_{ISO}\\ C_{CLVD}\\ C_{DC} \end{array}\right]=\frac{1}{M}\left[\begin{array}{l} M_{ISO}\\ M_{CLVD}\\ M_{DC} \end{array}\right]$$

(4) $$M=\left|M_{ISO}\right|+\left|M_{CLVD}\right|+M_{DC}$$

Based on the values of *C_ISO_*, *C_DC_*, and *C_CLVD_*, the mechanical types of rockburst events can be classified into Compression-dominated, Shear-Compression, Shear-dominated, Shear-Tension, Tension-dominated, Implosion-dominated, and Explosion-dominated. The identification criteria are shown in Table 2 [32]:

Meanwhile, to further investigate the distribution characteristics of rockburst event source locations in the vertical direction, and considering the low vertical positioning accuracy of existing MS monitoring systems, this paper corrects them based on the moment tensor inversion theory of source mechanisms. After solving for the theoretical amplitude of the P-wave first motion displacement, the correlation coefficient (*R^2^*) between the theoretical and actual values at various vertical heights can be calculated using Equation (8). The depth corresponding to the maximum *R^2^* value is selected as the corrected vertical height of the seismic source.

(5) $$\bar{y}=\frac{1}{n}\sum_{i=1}^{n}y_{i}$$

(6) $$SS_{tot}={\sum_{i}\left(y_{i}-\bar{y}\right)}^{2}$$

(7) $$SS_{reg}={\sum_{i}\left(f_{i}-\bar{y}\right)}^{2}$$

(8) $$R^{2}=\frac{SS_{reg}}{SS_{tot}}$$

In the formula, *n* represents the number of geophones used for the source mechanism inversion of each mining rockburst event; *y_i_* is the measured P-wave first-motion amplitude recorded by each geophone; *f_i_* is the theoretical P-wave first-motion amplitude at each geophone for different vertical heights.

**Figure 5:**
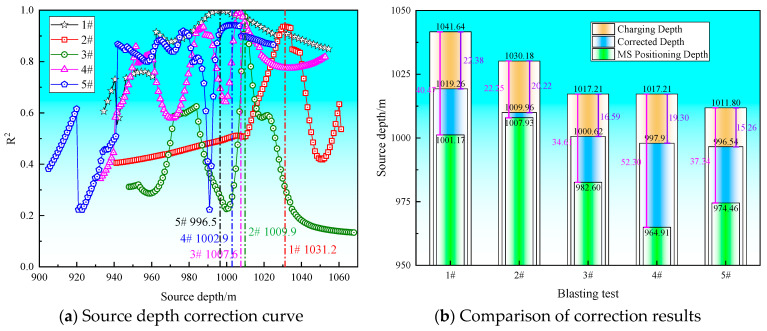
Validation of the source correction method.

To evaluate the effectiveness of the source depth correction method proposed in this study, five independent blasting tests were conducted in the field, with the depth correction curves and test results shown in Figure 5 and Table 3.

As illustrated in Figure 5, the sensor network layout in the 250101-2 working face is confined to a planar distribution. This geometric limitation typically results in insufficient constraint in the vertical direction, which leads to significant deviations between the vertical positioning results of the SOS MS monitoring system and the actual charging depths. In contrast, after applying the correction method proposed in this study, the positioning accuracy of the source depth for each blasting test was significantly improved, thereby validating the effectiveness and rationality of the method.

### 3.2. Source Rupture Type of Rockbursts

**Figure 6:**
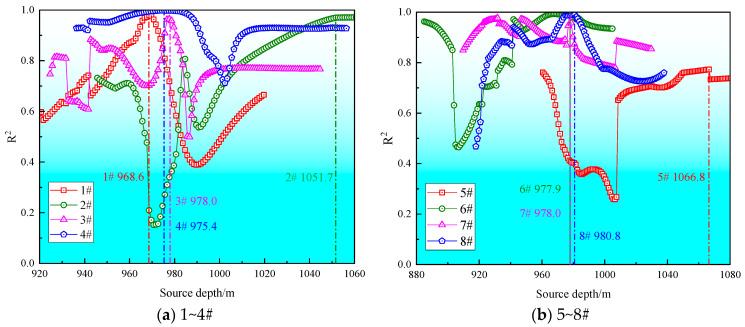
Correction curve for vertical seismic source positioning.

The calculation results of the correlation coefficients for the source locations of each rockburst event at various vertical heights are shown in Figure 6.

**Figure 7:**
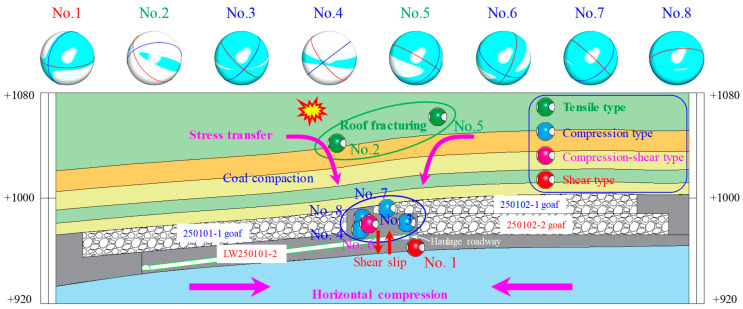
Schematic diagram of vertical seismic source distribution and rupture types.

Based on the borehole data from LW250101-2, a schematic cross-section was plotted as shown in Figure 7. Under the premise of disregarding the potential influences of monitoring network coverage and location errors on the spatial distribution of rockburst events, Projecting the corrected source coordinates onto Figure 7 reveals that five out of the eight rockburst events are located within the coal pillar zone, two are situated in the roof, and one is in the floor stratum. The source rupture types of the rockbursts in the coal pillar zone are exclusively compression failures, those in the roof are tension failures, and the event in the floor stratum is a shear failure. These distinct rupture types exhibit clear zonal distribution characteristics, corresponding to different rockburst failure mechanisms. This indicates the complexity of the failure mechanisms in the LW250101-2 haulage gateway, which are influenced by multiple factors.

Specifically, the compression failures are caused by the violent crushing of the coal pillar under high stress. The residual coal pillar belongs to the upper slice of the coal seam. According to burst tendency tests, the upper slice coal has a relatively high uniaxial compressive strength and possesses a strong burst tendency. Consequently, the energy released during coal crushing is significant. The tension failures result from the tensile fracture of the overlying roof strata, which is associated with the intense strata movement induced by the mining of the ultra-thick coal seam. In situ stress measurements indicate that the maximum principal stress in the 2501 mining district of Huating Coal Mine is the horizontal stress, oriented perpendicular to the roadway axis. Therefore, the shear failure is caused by the combined action of the maximum horizontal stress and the high vertical stress transferred from the coal pillar, leading to shear failure in the floor stratum. Compared to compression and tension failures, shear failures release more energy and possess greater disaster potential [33]. Observations from the field damage conditions confirm this conclusion.

## 4. Mechanism Analysis of Rockburst in Staggered Roadway

### 4.1. Numerical Model Establishment

**Figure 8:**
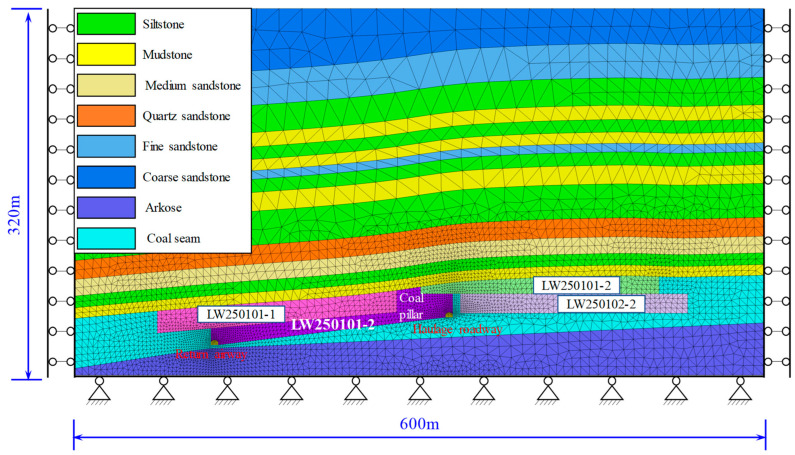
Mesh generation diagram of the FLAC^3D^ numerical model.

Based on the occurrence conditions of the strata in Huating Coal Mine, a simplified FLAC^3D^ numerical model was established using Rhino 6.0 software, as shown in Figure 8. The model dimensions are 600 m × 600 m × 320 m (X × Y× Z). The established model encompasses four working faces: LW250101-1, LW250102-1, LW250101-2, and LW250102-2. The widths and burial depths of the working faces were configured based on actual parameters. The vertical stress was generated by the self-weight of the strata, while the horizontal stress was set to 1.5 times the vertical stress according to the in situ stress measurement results. The Mohr-Coulomb model was selected as the constitutive model, and the mechanical parameters of the coal seams and rocks are listed in Table 4.

To effectively capture the fracture process of the rock strata during FLAC^3D^ simulations, interfaces were installed between the roof strata, and the large-strain mode was activated. The parameters for the interfaces were calculated using Equation (9):

(9) $$k_{n}=k_{s}=\left(K+\frac{4}{3}G\right)/\Delta z_{\min}$$

In the formula, *k_n_* represents the normal stiffness of the interface; *k_s_* represents the shear stiffness of the interface; *K* is the bulk modulus; *G* is the shear modulus; and $\Delta z_{\min}$ is the smallest width of the zone adjacent to the interface in the normal direction.

### 4.2. Characteristics of Stress Evolution

**Figure 9:**
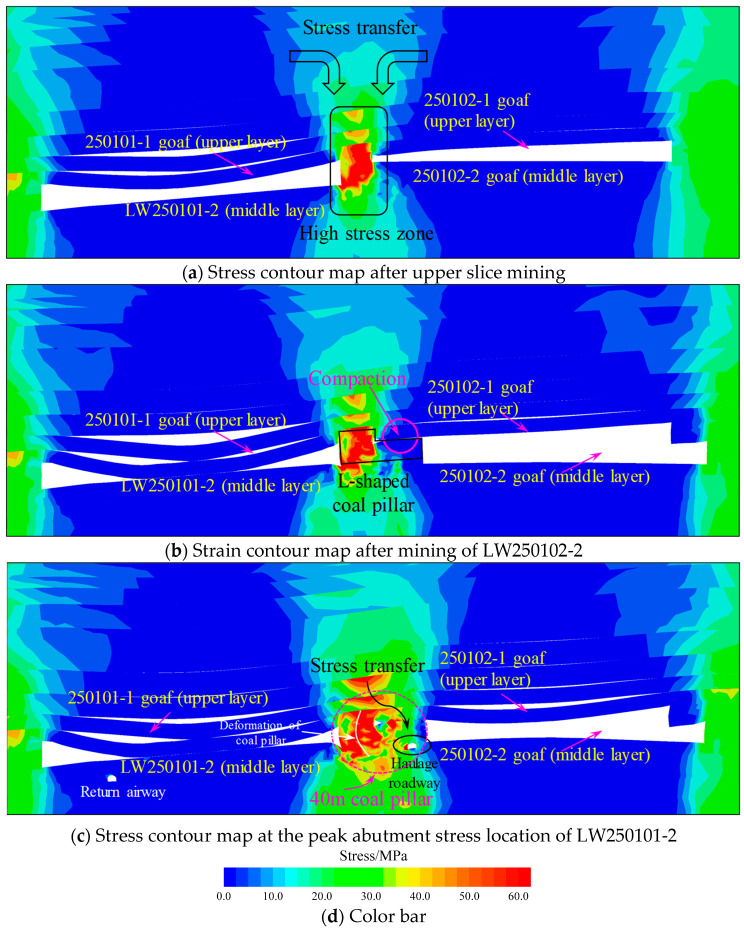
Stress evolution contour map.

The mechanism of rockburst occurrence is complex. Based on previous research results and field experience, high-stress zones correspond to a high risk of rockbursts [34,35]. Therefore, analyzing the stress evolution during the mining process of the working face can effectively reveal the causes of rockburst events. Figure 9 shows the contour map of stress evolution during the simulated mining process.

Following the extraction of the upper slice, the stress was transferred to the residual coal pillar area due to the presence of goafs on both sides. The stress within the coal pillar radiates in an arc pattern towards the lower sections on both flanks. Furthermore, the relatively large width of the coal pillar provides significant load-bearing capacity, facilitating the formation of an elastic core zone within the pillar. At this stage, the extraction of the coal seam resulted in the subsidence of the roof strata. The smaller mining height on the left side of the LW250102-1 made this area more prone to compaction. After the extraction of the LW250102-2, the characteristic staggered layout of the upper and lower slices led to the combination of the small coal pillar left on the left side of LW250102-2 and the overlying residual coal pillar, forming an “L-shaped” coal pillar structure. Simultaneously, disturbed by the mining activities, the roof strata further collapsed above the right wing of the “L-shaped” pillar and gradually compacted. During the mining process of the LW250101-2, the “L-shaped” coal pillar transitioned into a 40 m wide rectangular coal pillar. The haulage gateway is situated precisely within this pillar. At this point, the width of the stress transmission channel has significantly increased, allowing the stress from the roof to be transferred downward from the compacted overburden strata to the surrounding rock area of the roadway. Additionally, the pressure from the roof caused bending deformation on the left side of the coal pillar. The internal stress transmission path within the pillar deflects to the right towards the floor side, further increasing the stress imposed on the haulage gateway.

**Figure 10:**
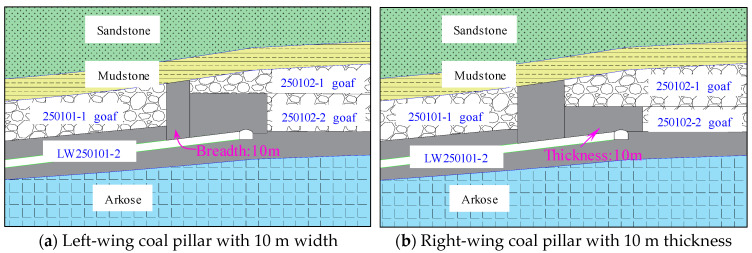
Schematic diagram of the control models.

**Figure 11:**
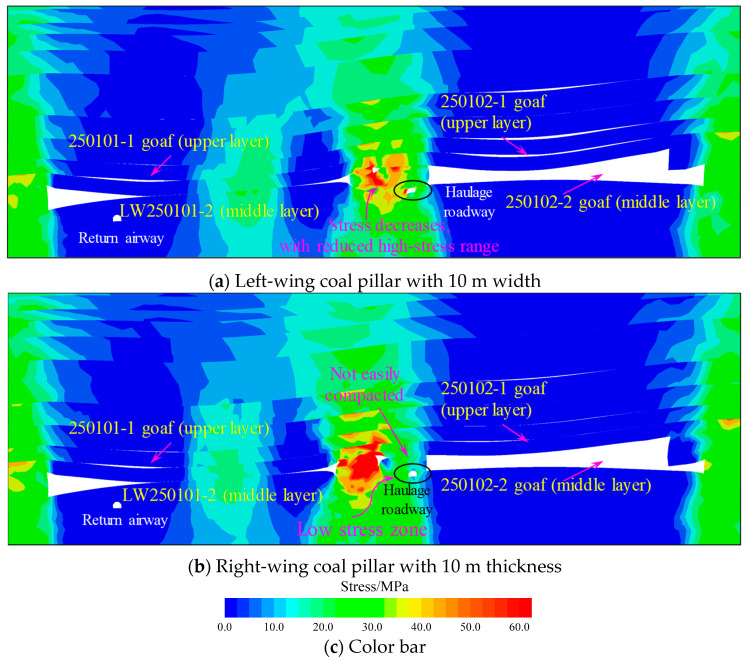
Stress evolution contour map under different controlling factors.

To further investigate the influence of the left-wing width and the right-wing thickness of the “L-shaped” irregular coal pillar on the LW250101-2 haulage gateway, two controlled models were established using the controlled variable method: one reducing the left-wing pillar width from 20 m to 10 m and the other reducing the right-wing pillar thickness from 18 m to 10 m. Schematic diagrams of these models are shown in Figure 10. The stress contour map at the peak abutment stress location of the LW250101-2 after following the aforementioned mining sequence is presented in Figure 11.

As shown in Figure 11a, when the width of the left-wing coal pillar decreases, the peak vertical stress in the pillar area is significantly reduced, accompanied by a substantial decrease in the extent of the high-stress zone. This phenomenon occurs because narrower pillars are more susceptible to plastic yielding, which reduces the elastic energy storage in the pillar zone through stress transfer effects. Meanwhile, smaller-sized pillars undergo progressive failure during the loading process. This mechanism facilitates continuous release of accumulated elastic energy within the coal mass, effectively preventing rapid stress concentration. The analysis demonstrates that the left-wing pillar width serves as a key controlling factor for rockburst manifestations in the LW250101-2 haulage gateway by regulating stress distribution patterns and energy accumulation levels.

As shown in Figure 11b, when the thickness of the right-wing coal pillar decreases (corresponding to an increased mining height on the left side of LW250102-1), the enlarged goaf space results in loosely compacted rock debris collapsing above the LW250101-2 haulage gateway. This collapse pattern significantly attenuates the vertical stress transmitted downward from the roof, creating a stress environment analogous to “below a goaf” where the surrounding rock experiences relatively low stress conditions. This analysis confirms that the right-wing thickness of the “L-shaped” pillar (mining height on the left side of LW250102-1) constitutes another critical factor influencing rockburst manifestations in the LW250101-2 haulage gateway.

### 4.3. Characteristics of Energy Distribution

The elements in the numerical model satisfy the Mohr-Coulomb yield criterion. The elastic strain energy *U* in the model’s coal-rock mass is calculated using the following formula [36]:

(10) $$U=\frac{1}{2E}\left[\sigma_{1}^{2}+\sigma_{2}^{2}+\sigma_{3}^{2}-2\mu \left(\sigma_{1}\sigma_{2}+\sigma_{1}\sigma_{3}+\sigma_{2}\sigma_{3}\right)\right]$$

In the formula, *E* represents the elastic modulus of the coal-rock mass; *σ*_1_ denotes the maximum principal stress of the coal-rock mass, *σ*_2_ the intermediate principal stress, and *σ*_3_ the minimum principal stress; *μ* is Poisson’s ratio. In FLAC^3D^, the rupture of rock mass is accompanied by a sudden release of energy. This process is manifested in the numerical model as a sharp drop in element stress and rapid release of strain energy. According to related research [37], during the model calculation, Fish language is used to execute Equation (10) at certain time steps. When the stress state of a unit reaches the yield condition and the stored elastic strain energy decreases, it is identified as an MS event. The energy of the MS events corresponds to the decrease in elastic strain energy of that unit, and the location of the event is recorded as the center coordinates of the unit.

**Figure 12:**
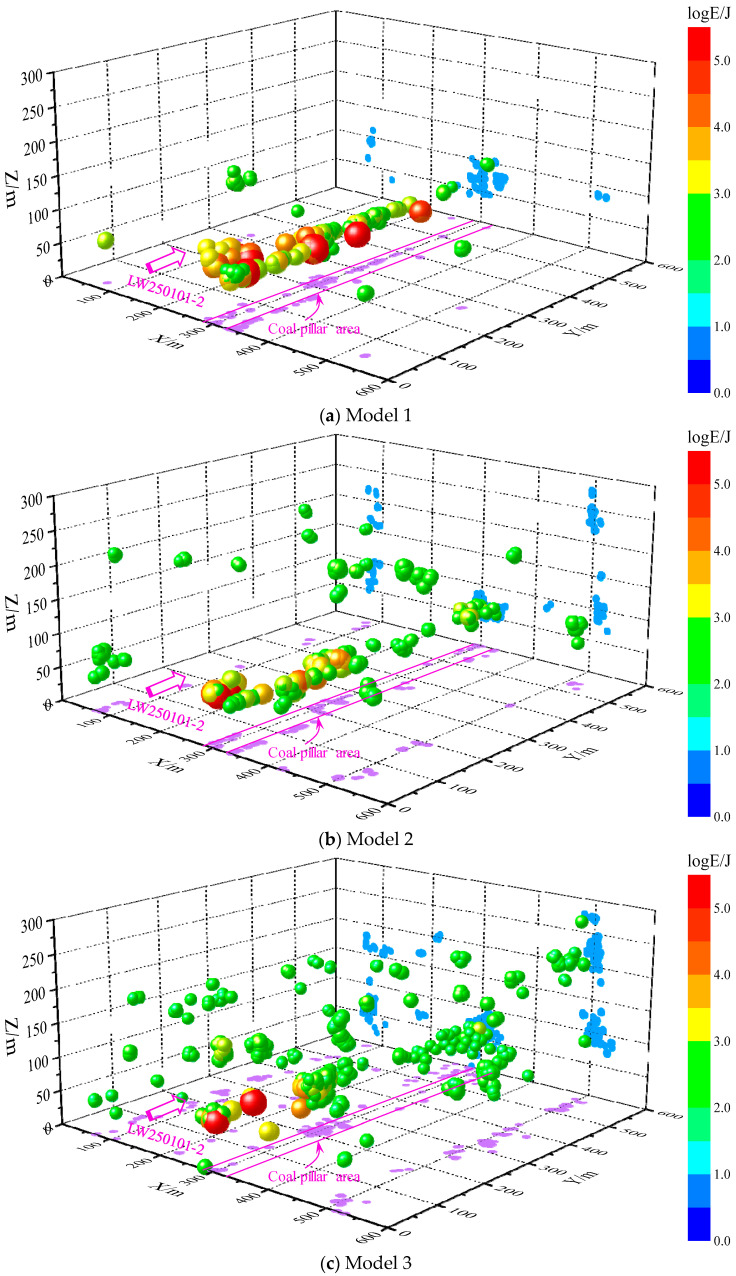
3D MS distribution map—Numerical simulation results.

**Figure 13:**
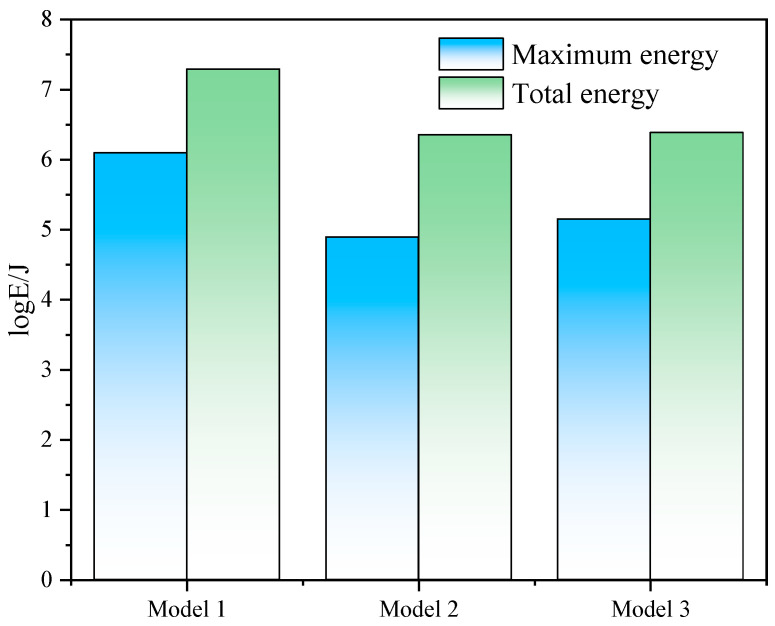
Energy Statistical Histogram of MS events.

To further validate the influence characteristics of coal pillar structures on rock bursts and clarify the impact mechanism of staggered roadway layouts under “L-shaped” irregular coal pillars, the occurrence of MS events after coal seam extraction in the three established models described above was compared. Among them, Model 1 represents the coal pillar structure under actual geological conditions, Model 2 involves reducing the width of the left-wing coal pillar to 10 m, and Model 3 entails reducing the thickness of the right-wing coal pillar to 10 m. The three-dimensional spatial distribution characteristics of MS events and the corresponding energy statistics generated by the three models are shown in Figure 12 and Figure 13.

As can be seen from Figure 12 and Figure 13, the numerical model constructed based on the existing geological conditions (Model 1) shows that after coal seam extraction, MS events are concentrated in the residual coal pillar area. This distribution pattern is consistent with the on-site monitored MS events, both exhibiting a typical banded distribution along the strike direction, demonstrating high consistency. Moreover, under this coal pillar structure, both the maximum energy and total energy of MS events are the highest. After altering the coal pillar structure (Model 2 and Model 3), although MS events still primarily occur in the coal pillar area, their concentration degree and energy magnitude are significantly reduced, with a noticeable trend of dispersion toward the roof. This characteristic of MS distribution aligns with the stress distribution results of the three aforementioned models, further indicating the control mechanism of coal pillar structure on rock bursts in staggered roadway layouts.

## 5. Discussion

### 5.1. Stress Deflection Effect

**Figure 14:**
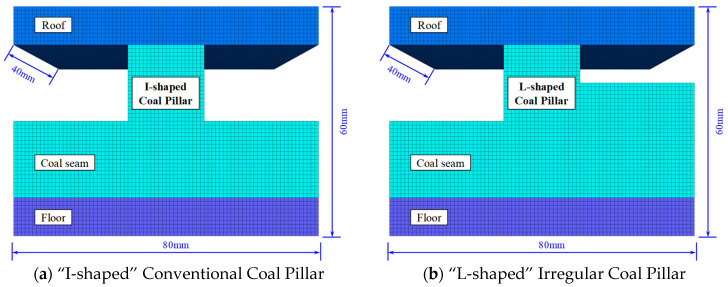
Coal-rock composite specimen model.

**Figure 15:**
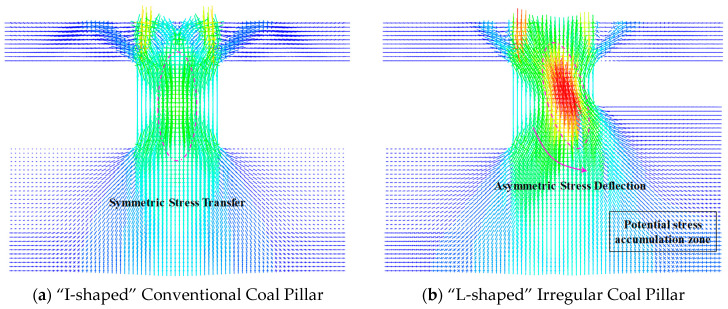
Vertical stress transfer path. (The dashed lines in the figure indicate the configuration and direction of stress).

As indicated by the numerical simulation results in Section 4.2, after the extraction of the upper slice and before the compaction of the roof above the right wing of the L-shaped coal pillar, the irregular geometry of the pillar leads to an asymmetric pattern of stress-transfer trajectories. To further reveal the fundamental differences in load-bearing mechanisms between the irregular L-shaped pillar and the conventional I-shaped pillar, and to investigate the influence of geometric morphology on the load-transfer path in the overlying strata, we simplified the complex engineering model into the coal-rock composite models shown in Figure 14. The overall dimensions of the models are set to 80 mm × 40 mm × 60 mm (length × width × height). Among them, Figure 14a represents the conventional I-shaped pillar structure, serving as the control group, while Figure 14b presents an L-shaped irregular pillar model with distinct geometric asymmetry. Figure 15 illustrates the stress transfer and deflection patterns in the two specimens after loading.

As shown in Figure 15a, the roof load is transferred vertically downward through the pillar to the floor, with stress streamlines evenly distributed and primarily concentrated in the central region of the pillar. The stress vector direction is essentially parallel to the vertical axis, exhibiting no significant lateral deflection. The high-stress concentration zone is located at the core of the pillar, presenting a symmetrically spindle-shaped distribution. This indicates that under the conventional “I-shaped” configuration, the surrounding rock pressure is mainly transformed into vertical compressive stress within the pillar, resulting in a relatively balanced overall stress state in the pillar. According to Figure 15b, due to the additional right-wing structure of the “L-shaped” pillar compared to the I-shaped pillar, the vertical stress trajectories undergo significant curvature. Simultaneously, the high-stress core is not located at the geometric center of the pillar but shifts toward the corner of the “L-shaped” configuration, displaying a certain rotational angle. This “stress deflection” phenomenon introduces additional horizontal shear components and bending moments, causing the pillar to not only bear vertical pressure but also to resist significant shear failure risks. This also corresponds to the compression-shear rupture type solved by source mechanism inversion. Under these conditions, the “L-shaped” pillar formed after the extraction of the upper-seam coal layer diverts roof stress toward the area below the right wing of the “L-shaped” pillar. Prior to the mining of the LW250101-2, the haulage gateway area had already become a potential stress accumulation zone.

### 5.2. Prevention and Control Measures and Effectiveness Verification

#### 5.2.1. Implementation Scheme of Prevention Measures

From the perspective of the mechanical properties and stress environment of coal and rock, the formation mechanism of rockburst can be attributed to the phenomenon where a coal-rock mass with burst tendency undergoes a dynamic failure under a specific stress state. Therefore, the physical properties of the coal-rock mass and its in situ stress environment are the key factors influencing rockburst. In other words, rockburst results from the interaction between the internal structure and mechanical properties of the coal-rock mass and the external stress environment. When subjected to sufficiently high pressure, the internal structure of the coal-rock mass may undergo changes, leading to its failure and deformation. Under certain stress conditions, this failure and deformation may manifest as a rockburst.

To effectively prevent and control the frequent occurrence of rockbursts in the LW250101-2 haulage gateway, techniques such as deep-hole blasting and large-diameter borehole pressure relief can be employed to weaken the stress environment of the surrounding rock. This technology is primarily applied to the coal pillar and roof, aiming to alter the stress distribution within the coal-rock mass, reduce the degree of stress concentration, or shift the peak stress zone away from the mining excavation, thereby fundamentally reducing the rockburst risk.

(1) Pressure relief by combined deep-hole blasting in roof

**Figure 16:**
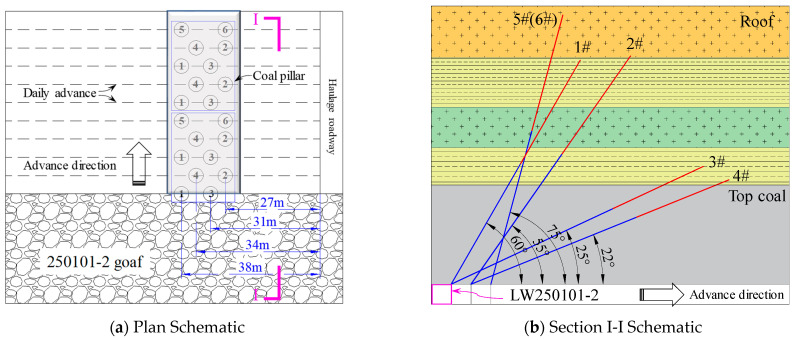
Schematic Diagram of Combined Deep-Hole Blasting Construction in Roof.

To effectively release and transfer the high stress in the overlying residual coal pillar, a high-low hole combination method was adopted to perform blasting treatment on the roof above the coal pillar. This measure can effectively shorten the breaking interval during the periodic weighting of the roof, promote more regular caving of the roof in the goaf, and weaken the continuous structure of the roof between the goaf and the area to be mined. Its core function is to reduce the dynamic loading induced by the sudden collapse of large-scale suspended roof strata at the working face [38]. The specific implementation plan is as follows: A total of 6 blasting boreholes were arranged, designated as #1, #2, #3, #4, #5, and #6. For every 2.4 m to 3.2 m of face advance, two roof deep-hole blasts were conducted according to the following sequence: #1, #3→#2, #4→#1, #3→#2, #4→#5, #6→#1, #3. The specific construction parameters are shown in Table 5, and a layout schematic is provided in Figure 16.

(2) Pressure relief by large-charge and deep-hole blasting in coal mass

**Figure 17:**
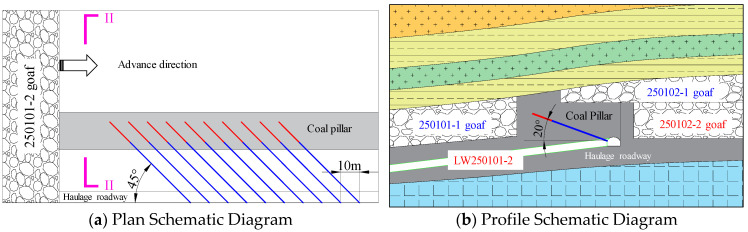
Construction schematic diagram of deep-hole blasting in roadway rib coal mass.

Implementing large-charge deep-hole blasting in the coal pillar ahead of the working face can significantly shift the stress concentration zone forward into the coal mass, thereby reducing the degree of superposition between dynamic loads during mining and the residual high-stress concentration zone. Its core effect lies in effectively mitigating the adverse influence of the overlying residual coal pillar on the stability of the surrounding rock on the side of the haulage gateway. Considering safety concerns related to rockbursts during construction, the blasting operations were conducted at a safe distance ahead of the working face. The specific implementation parameters are as follows: boreholes were drilled at a 20° upward angle and at a 45° angle to the working face’s tendency, with a depth of 53 m, a charge weight of 39 kg, and a charge length of 13 m. The hole-opening position was located on the rib of the haulage gateway, 2.2 m above the floor. The schematic diagram of the layout for large-charge and deep-hole coal blasting for pressure relief is shown in Figure 17.

(3) Pressure relief by ultra-deep and large-diameter boreholes

**Figure 18:**
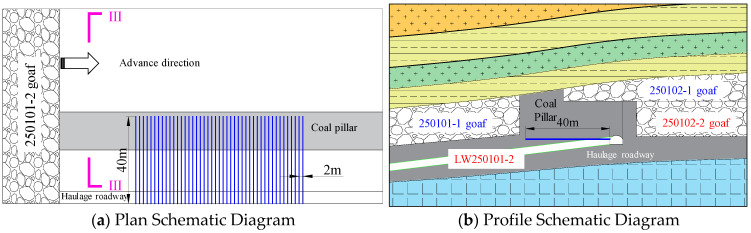
Schematic diagram of pressure relief by ultra-deep and large-diameter boreholes.

High in situ stress is the primary cause of the increased risk of rockburst disasters in deep mines. Large-diameter boreholes are a commonly used pressure relief method in high-stress zones. Therefore, a row of pressure relief boreholes was arranged on the solid coal side of the LW250101-2 haulage gateway. The boreholes had a depth of 40 m, a diameter of 250 mm, and a spacing of 2.0 to 2.5 m. The construction of these ultra-deep, large-diameter boreholes creates a certain compensation space within the coal mass. The compression of this compensation space enables the absorption of part of the high stress transferred from the floor of the overlying residual coal pillar to the LW250101-2 coal body, thereby reducing phenomena such as rib convergence and mesh cracking in the gateway. Simultaneously, the compensation space also helps to attenuate dynamic loads, effectively controlling the probability of dynamic load-induced rockburst disasters. The schematic diagram of the layout for ultra-deep and large-diameter pressure relief boreholes is shown in Figure 18.

#### 5.2.2. Effectiveness Evaluation Based on MS Data

**Figure 19:**
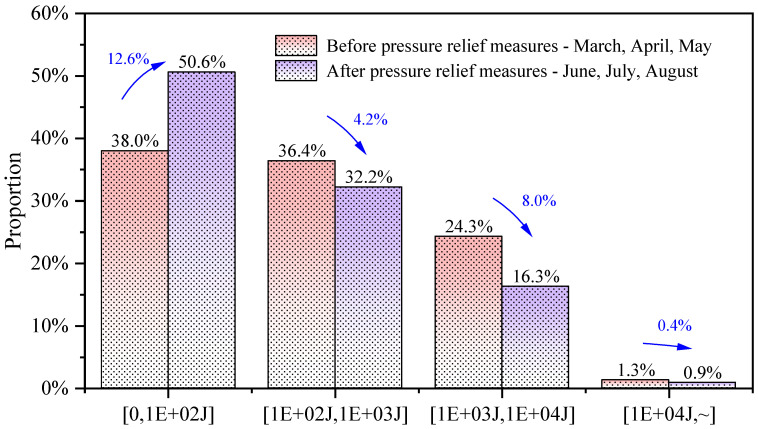
Comparison chart of MS events energy before and after implementing pressure relief measures.

To analyze the pressure relief effect of the aforementioned measures, MS data from equivalent time periods before and after the implementation of the pressure relief measures were selected. The proportion of MS events in each energy range was statistically analyzed and compared, as shown in Figure 19. The statistical results indicate that after the implementation of the measures, the proportion of MS events in the [0, 1 × 10^2^ J] energy range increased significantly, while the proportions in all other energy ranges decreased. This shows that after the measures were applied, energy release is dominated by small-energy events, suggesting that the current stress state of the rock mass is more dispersed, substantially reducing the risk of rockburst.

**Figure 20:**
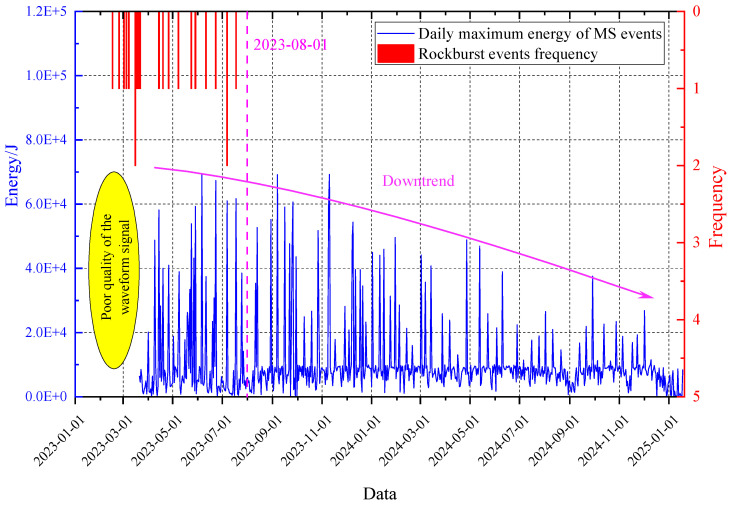
Evolution curve of rockburst frequency and daily maximum energy.

To evaluate the pressure relief effect over the entire mining period, evolution curves of rockburst frequency and the daily maximum energy of MS events were plotted, as shown in Figure 20. The figure indicates that after the implementation of rockburst prevention measures in June 2023, both the frequency of rockburst manifestations and the energy released significantly decreased. Furthermore, the occurrence of rockbursts was essentially eliminated by August 2023. Subsequently, after November 2023, the daily maximum energy of MS events showed a notable declining trend until the safe conclusion of mining at the working face.

### 5.3. Optimization of Working Face Design

**Figure 21:**
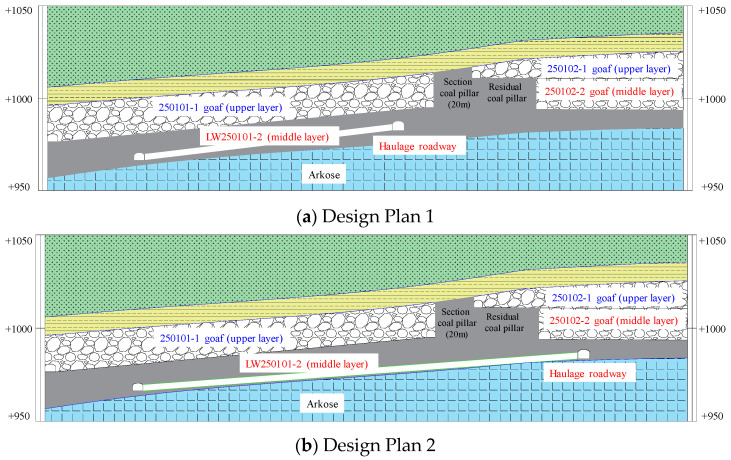
Optimized Working Face Design Plan.

Previous studies in this paper indicate that the LW250101-2 haulage gateway is located in a high-stress concentration zone due to the influence of the “L-shaped” structure. On-site observations show that the source locations of rockburst events and the dense areas of high-energy MS activity are entirely situated on the coal pillar side of the haulage gateway. Therefore, in the subsequent design of the working face mining gateway, it should be kept away from the high-stress concentration zone, as shown in Figure 21.

## 6. Conclusions

Based on MS data analysis, numerical simulation, and source mechanism inversion, this study investigated the rockburst mechanism and prevention techniques for staggered roadways under the influence of overlying wide residual irregular coal pillars in ultra-thick coal seams, using the LW250101-2 in Huating Coal Mine as the engineering background. The main conclusions are as follows:

(1) In response to the problem of limited vertical positioning accuracy due to the planar layout of seismic networks, a vertical height correction method for seismic sources based on the moment tensor mechanical mechanism was proposed. Five sets of blasting tests were conducted to verify the effectiveness of the method. Combining the results of seismic source mechanism inversion, the rupture types of rockburst events were further clarified. Coal pillar compression failures accounted for the highest proportion, followed by roof tension failures. The source rupture type is primarily controlled by factors such as coal burst tendency, movement of the roof overburden, and the magnitude of horizontal stress.

(2) According to numerical simulation results, the overlying section coal pillar and the underlying residual coal pillar form an “L-shaped” high-stress structure. The left-wing width and the right-wing thickness (adjacent slice mining height) of this “L-shaped” irregular coal pillar jointly determine the rockburst risk level of the LW250101-2 haulage gateway. A larger left-wing width and a greater right-wing thickness (lower adjacent slice mining height) correspond to a higher rockburst risk in the LW250101-2 haulage gateway. Furthermore, compared to conventional “I-shaped” coal pillars, the stress deflection effect induced by the “L-shaped” pillar causes the haulage gateway of LW250101-2 to remain in a zone of sustained stress accumulation, thereby increasing the risk of rockbursts. Therefore, during slice mining of ultra-thick coal seams, wide section pillars should be avoided in the upper slices. Simultaneously, roadways within the same slice should ideally be positioned at the same elevation to eliminate the right-wing structure of the “L-shaped” pillar and reduce the rockburst risk.

(3) Prevention and control measures for rockbursts under the influence of overlying irregular coal pillars were proposed. Based on MS data collected during the mining period, the energy distribution of MS events and the occurrence of rockbursts before and after the implementation of these measures were compared and analyzed. The results show that the implemented measures, including deep-hole blasting in the roof and coal mass and large-diameter borehole pressure relief in the coal mass, effectively prevented the occurrence of rockbursts and ensured the safe mining of the working face.

## Figures and Tables

**Table 1:** In situ stress test results.

Station No.	Depth (m)	*σ*_1_			*σ*_2_			*σ*_3_		
		Value	Direction	Dip Angle	Value	Direction	Dip Angle	Value	Direction	Dip Angle
		(MPa)	(°)	(°)	(MPa)	(°)	(°)	(MPa)	(°)	(°)
1#	685	33.04	252	8	22.32	345	18	18.39	320	−70
2#	480	22.34	254	17	13.96	67	73	13.65	343	−2

**Table 2:** Corresponding relationship between source type and moment tensor component.

Type	*C_DC_*	*C_ISO_*	*C_CLVD_*	|*C_ISO_*| + |*C_CLVD_*|
Compression-led type	*C_DC_* < 10%	*C_ISO_* < 0	*C_CLVD_* < 0	|*C_ISO_*| + |*C_CLVD_*| ≥ 90%
Compressive-shear type	10% ≤ *C_D__C_* < 60%	*C_ISO_* < 0	*C_CLVD_* < 0	40% < |*C_ISO_*| + |*C_CLVD_*| ≤ 90%
Shear-led type	60% ≤ *C_DC_*	/	/	|*C_ISO_*| + |*C_CLVD_*| ≤ 40%
Tensile-shear type	10% ≤ *C_DC_* < 60%	*C_ISO_* > 0	*C_CLVD_* > 0	40% < *C_ISO_* + *C_CLVD_* ≤ 90%
Tensile-led type	*C_DC_* < 10%	*C_ISO_* > 0	*C_CLVD_* > 0	*C_ISO_* + *C_CLVD_* ≥ 90%
Implosion-led type	/	*C_ISO_* < 0	|*C_ISO_*| ≥ 90%	/
Explosion-led type	/	*C_ISO_* > 0	*C_ISO_* ≥ 90%	/

**Table 3:** Comparison of correction results.

Blasting Test	Charging Depth (m)	MS Positioning Depth (m)	MS Positioning Depth Absolute Error (m)	Corrected Depth	Corrected Depth Absolute Error (m)	Reduction in Error (%)
1#	1041.64	1001.17	40.47	1019.26	22.38	44.70%
2#	1030.18	1007.93	22.25	1009.96	20.22	9.12%
3#	1017.21	982.60	34.61	1000.62	16.59	52.07%
4#	1017.21	964.91	52.30	997.91	19.30	63.10%
5#	1011.80	974.46	37.34	996.54	15.26	59.13%
MS Positioning Depth Mean Absolute Error (m)			37.39	Corrected Depth Mean Absolute Error (m)		18.75
MS Positioning Depth Mean Absolute Error (m)			10.83	Corrected Depth Mean Absolute Error (m)		2.85

**Table 4:** Mechanical parameters of coal and rock mass.

Formation	Bulk Modulus (GPa)	Shear Modulus (GPa)	Cohesion (MPa)	Tensile Strength (MPa)	Internal Friction Angle (°)	Density (kg/m^3^)
Coarse sandstone	4.68	2.29	3.27	2.13	28.87	2040
Mudstone	4.49	1.84	2.10	2.27	29.85	2350
Medium sandstone	3.67	2.20	4.82	2.07	20.18	1930
Quartz sandstone	7.56	4.76	7.24	2.67	30.71	2170
Fine sandstone	4.90	2.86	4.13	2.00	29.21	2420
Siltstone	4.93	2.96	4.73	3.56	24.36	2530
Coal seam	1.71	0.25	1.21	2.22	30.44	1280
Arkose	3.52	2.83	1.96	2.14	28.43	2478

**Table 5:** Blasting construction parameters.

Hole No.	Hole Diameter /mm	Inclination /°	Hole Depth /m	Charge Column Length /m	Charge Weight /kg
1#	75	60	65	30	90
2#	75	55	70	30	90
3#	75	25	70	25	75
4#	75	22	70	25	75
5#	75	75	70	30	90
6#	75	75	70	30	90

## Data Availability

The raw data supporting the conclusions of this article will be made available by the authors on request.
